# Daily time management in dementia: qualitative interviews with persons with dementia and their significant others

**DOI:** 10.1186/s12877-023-04032-8

**Published:** 2023-07-03

**Authors:** Ann-Christine Persson, Lena Dahlberg, Gunnel Janeslätt, Marika Möller, Monika Löfgren

**Affiliations:** 1 Department of Clinical Sciences, Danderyd Hospital, Karolinska Institutet, 182 88 Stockholm, Sweden; 2 Department of Rehabilitation Medicine, Danderyd University Hospital, Entrévägen 8, plan 2, 182 88 Stockholm, SE Sweden; 3 School of Health and Welfare, Dalarna University, 791 88 Falun, Sweden; 4 Aging Research Center, Karolinska Institutet & Stockholm University, 18A, 171 65 Tomtebodavägen, Solna, Sweden; 5 Department of Public Health and Caring Sciences, Uppsala University, Uppsala, Sweden; 6 Center for Clinical Research in Dalarna, 791 29 Box 712, Falun, SE Sweden

**Keywords:** Agency, Assistive technology, Informal care, Older adults, Participation, Strategies, Qualitative research

## Abstract

**Background:**

Persons with dementia encounter time-related problems and significant others often need to provide support in daily time management and use of time assistive technology (AT). Further research has been requested on how time AT for persons with dementia affects the situation of significant others. Moreover, there are a few previous qualitative studies on the experiences of time AT by persons with dementia. This study explores the experiences of persons with dementia and significant others in daily time management and their perceptions on how time AT affects everyday life.

**Method:**

Semi-structured interviews with persons with mild to moderate dementia (*n* = 6) and significant others (*n* = 9) were conducted three months after receiving prescribed time AT. Interviews were analysed using qualitative content analysis.

**Results:**

A main category “*Support by significant others is always part of daily time management*”, and three categories “*Facing new challenges*”, “*Using strategies to handle changes*”, and “*Time assistive technology in daily life*” illustrated that significant others provided support for daily time management in all phases of dementia. This support was often embedded in other kinds of support for emerging challenges. Support in time management was needed from an early stage in dementia, and responsibility for time management was gradually transferred to significant others. Time AT could support time orientation and were important for sharing the time management conducted by others but did not enable independent time management.

**Conclusions:**

Time-related assessments and interventions should be offered at an early stage of dementia to increase the possibility of maintaining daily time management skills. Using time AT to communicate time might increase agency and participation in daily occupations for persons with dementia. Given the central role of significant others for daily time management, the society needs to adequately support persons with dementia lacking support from significant others.

## Introduction

Dementia is one of the most common global conditions. In the world, there are about 47 million persons living with dementia and in 2030 the estimated number is 75 million due to aging populations [1]. Alzheimer’s disease is the most common form of dementia, followed by vascular dementia. Other common types of dementia are frontotemporal dementia, dementia with Lewybodies and mixed dementia [2]. Dementia often affects time-related functions and the ability to independently manage time in daily life [3, 4]. Persons with dementia might experience problems with missed appointments, too early or too late arrivals, and uncertainty when something is going to happen. Problems understanding clocks, knowing how much time is left of an activity and estimating the time required when planning activities are other examples of time-related difficulties affecting daily occupations. Daily occupation in this context refers to anything that persons do in their daily lives and includes not only a single activity but also a set of activities together [5]. It might also be difficult to carry out a daily routine; for example to take medication at the right time or to eat regularly [6]. Strategies or environmental support can be used in daily time management; e.g. looking at the date on a newspaper to keep track of days and dates or setting reminders to start necessary preparations to be ready in time to time-specific events [6, 7]. Everyday technology, like coffee machines with automatic timers or electronic calendars in smart phones can also provide support in daily time management. However, strategies may not be effective [6, 7] and there is a risk that everyday technology might become too difficult to use as cognition deteriorates [8]. Over time, dementia leads to increasing need for support in daily time management; support that is often provided by significant others, such as partners, family members, or friends [3, 9]. In addition, time assistive technology (AT) is often prescribed to persons with dementia. [6, 10, 11]. This study will focus on experiences and perceptions of persons with dementia and their significant others, more specifically on their experiences of daily time management and perceptions as to how time AT affects their everyday life.

Previous research has found that persons with dementia can benefit from time AT [8, 12–14]. The use of AT for time orientation could, for example, lead to increased independence, less anxiety, and fewer questions about the time of the day [15]. Research has also shown that time reminders are effective in supporting persons with cognitive impairments [16, 17]. In Sweden, AT can be prescribed through special centres with a set range of products, although products available may vary across regions and municipalities. The greater part of prescribed AT is paid by taxes, but each region and municipality decide on its own fees. Prescribers are usually health care professionals, such as occupational therapists [18]. In this study, the general term “time AT” is used for AT prescribed for support in daily time management by compensating for impairments in orientation to time, experience of time, and time management.

However, for persons with dementia to be able to use and benefit from AT, support from significant others is important [7, 13, 19, 20]. In Sweden, most persons with dementia live in ordinary housing [3], receiving help from their significant others, such as family and close friends [21]. A study exploring occupational therapists’ experiences of prescribing time AT found that significant others played an important role in whether, and in which ways, the device was implemented into the daily routines of the persons with dementia [6]. Systematic reviews investigating AT for older adults, including persons with dementia, has found that AT might both decrease significant others’ time spent, effort, and level of support, and increase independence for persons with dementia [22, 23]. However, evidence regarding relief for significant others is conflicting as research has also shown that AT could increase significant others’ burden and stress levels, for example, when the use of AT seemed to increase the dependence of the person with dementia on the significant other [22–25]. Adapting and maintaining AT use for persons with dementia often involves learning, problem-solving, and seeking technical support [26]. Significant others may face problems in supporting the person with dementia in using the device, due to poor comprehension on how the device works, poor device design, lack of support from professionals in implementing AT use, and lack of knowledge of available AT [23]. Another issue might be that significant others’ needs, attitudes, knowledge, expectations, and experiences of AT might differ from those of the persons with dementia [25, 27]. Further research as to how time AT for persons with dementia affects significant others’ situations have been requested [22, 23].

Still, the perspectives of significant others are dominating in research on technology use for persons with dementia [25], although the importance of including persons with dementia has been emphasised [13, 28]. There are a few studies on experiences of time AT of persons with dementia, showing that motivation, insight, adjustment to routines, improved capacity and available support from another person are essential if a new device is to be successfully implemented [13, 15, 29]. Gaining further insights into individual experiences on daily time management and how to support persons with dementia and their significant others through time-related interventions, such as time AT, are important in clinical research and healthcare settings and can contribute to the development of improved and targeted time-related assessments and interventions. Altogether, it is important to increase knowledge on how older persons with dementia manage time in daily life including the use of strategies; support from significant others; the process of receiving and implementing time AT; and how time AT impacts daily life, based on individual and common experiences of persons with dementia and significant others. Thus, the aim of this study was to explore the experiences of persons with dementia and significant others regarding daily time management and their perceptions on how time AT affects their everyday life.

## Methods

### Design

This interview study has a qualitative design. The current study is part of the research project “Managing time with dementia”. The aim of the project is to investigate and model the relationship between the use of time AT by older persons with mild to moderate dementia, their daily time management, and their well-being, and to devise strategies for the acceptance and use of such products by older persons with dementia and significant others.

### Participants

Persons with dementia and significant others participated in the study. Inclusion criteria for persons with dementia were diagnosed dementia, ≥ 11 on the Mini-Mental State Examination (MMSE) [30], daily time management problems identified by occupational therapists in memory assessments, receipt of time AT prescribed by the occupational therapist at least three months prior, ability to communicate in Swedish, and no mental illnesses unrelated to dementia. Inclusion criteria for significant others included knowledge about the person with dementia’s daily life, daily time management and usage of time AT ≥ three months, and ability to communicate in Swedish. With a purposive sampling approach, variation in recruitment was sought in terms of gender, relationship (partner, child, friend), family situation (single living alone, partner not living together, partner/family living together), and prescription of easy-to-use or advanced time AT. Persons with dementia and significant others could participate in the study even if their close one chose not to participate. In this study there were six couples, and three significant others that participated without the person with dementia. Participant demographics are presented in Table 1. All participants but one were spouses living together with another person, and seven out of nine significant others provided daily support. Two significant others provided support between two and six times a week.

**Table 1:** Characteristics of persons with dementia and significant others, interview type, and time assistive technology

	Persons with dementia (*n* = 6)	Significant others (*n* = 9)
Gender (*n*)		
Women	3	6
Men	3	3
Age, years (md, range)	75 (71–77)	69 (34–73)
Family situation (*n*)		
Spouse, living together	6	8
Parent/child, not living together		1
Prescribed time assistive technology for person with dementia (*n*)		
Electronic calendar, date, day	1	1
Electronic calendar, date, time, day, night	5	6
Weekly board and voice note recorder	1	1
Time management support with reminders		2
Interview mode (*n*)		
Alone, face-to-face interview		1
Alone, telephone interview	3	5
Dyad, telephone interview	1	1
Dyad, video-based interview	2	2

Type of prescribed time AT occurring in the study: (a) an easy to use electronic calendar displaying the date and day; (b) an easy to use electronic calendar displaying information about date, time, and whether it is morning, daytime, evening, or night; (c) a magnetic weekly board for time management to be used in combination with a device for recording of voice notes; and (d) an advanced time management support device showing what day it is, the time of the day, activities scheduled for the day, week, and month, with the possibility of including time reminders and checklists. The frequency of each time AT is reported in Table 1.

### Procedure

Separate interview guides with open-ended questions for persons with dementia and significant others were developed by the research group, where short questions with clear language were used to facilitate the participation of persons with dementia. The interview guide for significant others included additional questions about providing support in using time AT. The development of the interview guides was based on a previous study investigating occupational therapists’ experiences of prescribing time AT for persons with dementia [6], and on previous research in which AT was developed and adjusted to the needs of cognitive impairments [31, 32]. The semi-structured interview guides covered the following topics: daily time management problems, daily time management strategies, prescription, implementation and follow-ups of time AT, experiences of using/supporting the use of time AT, and the impact of time AT use on everyday life. A pilot interview was conducted to evaluate and revise an early version of the interview guide for significant others. The pilot interview was included in the study.

Thirteen occupational therapists working on memory investigations at 11 memory clinics recruited participants and collected data for the main project. Persons who were eligible for interviews during 2020 and 2022 were asked whether the first author could contact them by phone with information about the interview study and an invitation to participate. Possible participants also received written information about the study. Everyone that agreed were included in the study. Finally, six persons with dementia and nine significant others were recruited via eight memory clinics. The clinics were located in the middle part of Sweden, covering both urban and rural areas.

Persons with dementia and their significant others were interviewed by the first author, individually or together depending on participants’ wishes. Time and length of the interviews, and the way in which the interviews were conducted (individual or dyad) were flexible and based on the wishes of each participant.

In total, twelve interviews lasting 15–60 min (mean 29 min) were conducted with 15 participants. Based on prevailing Coronavirus disease of 2019 (COVID-19) restrictions, interviews were carried out by phone, via video calls, or face-to-face (Table 1). Demographic data for persons with dementia and the significant others was collected during data collection for the main project. Interviews were digitally recorded (audio) and transcribed verbatim.

### Data analysis

**Fig. 1:**
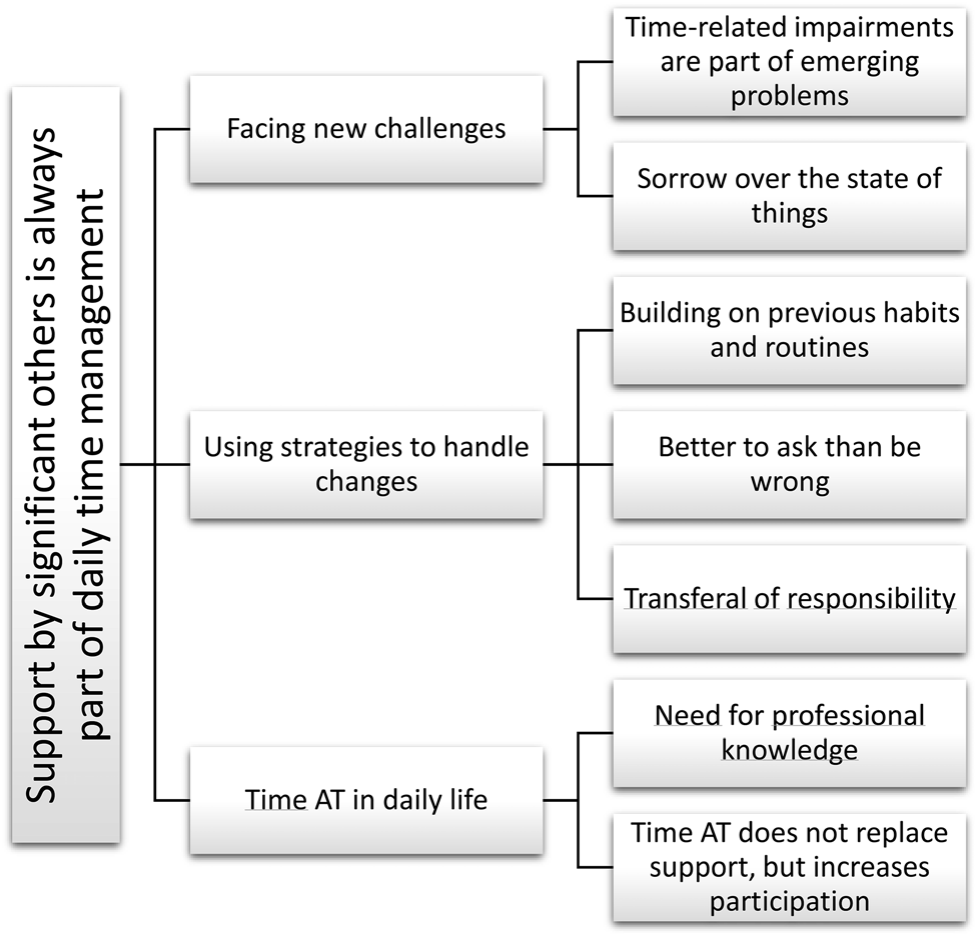
Main category, categories, and subcategories. Examples of codes in foot notes. ^1^Uncertainty about when and where things happen; In need of support with time management from significant other; Mixing up the days. ^2^Experiencing a defeat of not being able to plan time; Difficult to accept changes. ^3^Previously important to keep track of time; Previous habit of using calendar. ^4^Uses calendar but still asks significant other about time; A need of confirmation of time management. ^5^Significant other keeps track of when things happen; Handing over responsibility instead of asking. ^6^No previous knowledge of time AT; Would have liked to receive time AT earlier; ^7^Time AT reduce the need of questions; Takes part in time planning with time AT

Interviews were analysed using the principles for qualitative inductive content analysis [33]. An emergent design was used; data analysis was conducted in parallel with data collection [34]. The emerging results were used to further develop the interview questions. The analysis started with reading through the transcriptions several times to get an overall understanding of the material. Next, coding close to the data and groupings of the codes were made to create preliminary subcategories for comparison based on similarities and differences. After this, the subcategories were compared and clustered until preliminary categories emerged. This process was carried out for each interview separately before merging the findings from all interviews into preliminary sub-categories, categories, and one main category (Fig. 1). The text was coded in the Open Code software programme (https://www.umu.se/en/department-of-epidemiology-and-global-health/research/open-code2).

Several actions were taken to enhance the trustworthiness, including corroboration, triangulation, peer debriefing and disclosure. Corroboration was done through constant comparison of all codes and categories with the interviews to make sure that analysis was grounded in the data. After fifteen interviews, no new information that differed from that gained through preceding interviews was obtained and data collection was ended. Triangulation was used for sources of data. Both persons with dementia and significant others were interviewed, covering different perspectives on daily time management and time AT. Triangulation by researchers with different perspectives was used to increase credibility: AP is an occupational therapist with experience in cognitive rehabilitation research; LD has extensive experience in social gerontological and social work research; GJ is an occupational therapist with extensive research experience in time-related assessments and interventions; MM is a neuropsychologist with extensive experience in dementia research; and ML is a physiotherapist with extensive experience in qualitative methodology. AP coded all interviews, and ML and LD coded parts of the interviews separately for comparisons and discussions. AP, ML and LD held regular meetings throughout the analysis process to discuss emerging codes and categories until agreement was reached, and then findings were discussed in detail with all authors. Peer-debriefing of preliminary results were done with an experienced clinician and at an OT seminar. To further increase trustworthiness and transparency, disclosure was sought through clear presentation of the analysis method, and interview numbers allocated to each citation of the original data in the presentation of the results [34].

## Results

The analysis of the interviews revealed a main category: *Support by significant others is always part of daily time management*, and three categories: *Facing new challenges*, *Using strategies to handle changes*, and *Time AT in daily life*, each including two or three subcategories (Fig. 1). Below, results are presented separately for persons with dementia and significant others, by category.

### Main category: support by significant others is always part of the daily time management

The overarching finding represented by the main category illustrated that significant others provided support for daily time management in different situations in all phases of dementia, from emerging problems to maintaining of habits and routines. Gradually, overall responsibility of daily time management was transferred to the significant other. Although easy-to-use time AT supported time orientation, it did not replace support from significant others, since the implementation was dependent on support from them and since the person with dementia needed further support to put time into context. Such information could be provided via calendars and advanced time AT, but except from the need of support to carry out time management and handle the device, persons with dementia also experienced a need to confirm the scheduled information with their significant other.

## Category 1: facing new challenges

### Time-related impairments are part of emerging problems

*Participants with dementia* described a variety of emerging problems related to dementia, such as memory loss, fatigue, activities taking longer time to perform than before, and feelings of uncertainty about when and where things were going to happen. Even though life as a pensioner no longer entailed the same need to keep track of time as had been the case when working, disorientation in regard to time and day was experienced as problematic. Daily time management could no longer be carried out independently, and the use of everyday technologies—such as smart phones or electronic calendars—became more and more difficult. Participants with dementia also described increasing memory problems leading to mistakes, which, in turn, created anxiety about making new mistakes. Persons with dementia described how low confidence in one’s own ability led to avoidance of activities, rather than risking failures, especially when it came to economy and paying bills.

Persons with dementia experienced that it was tough to notice, acknowledge, and adapt to problems caused by dementia, and to give up previous occupations that could no longer be carried out. Not being able to act like before affected their participation in social contexts, and it was uncomfortable to notice other persons’ reactions to problems in remembering or finding the correct words:

*“And that’s a spontaneous reaction that most people have, they roll their eyes, like yeah, what is it?“* [11]

*Significant others* also described problems affecting daily time management as a part of emerging dementia-related challenges.

*“But it’s clear that time plays a big part in dementia itself. The issues around time are a big part of it. When and where and how and so on.“* [6]

The significant others in the study reported that persons with dementia needed support in carrying out daily time management, although it varied as to whether the significant others considered it to be a major problem. Other daily challenges mentioned included memory impairments, problems related to orientation in place, the constant need to look for misplaced objects, the lack of insight, and difficulties in learning.

Moreover, the significant others considered that stress and mood changes exacerbated already-existing difficulties for persons with dementia. Conversely, decreased control of time could also create feelings of stress. Participants interviewed during the COVID-19 pandemic described how restrictions contributed to a shrinking world and diminished the need for time management for activities outside the home and for social events.

### Sorrow over the state of things

*Persons with dementia* found it troublesome to no longer being able to take care of oneself due to dementia. The loss of independence created feelings of inadequacy, low self-confidence, and insecurity about their capability of performing daily occupations. It felt like a defeat to no longer take care of oneself or participate in daily planning, including daily time management.

*“...at the same time, you feel unsure about things, so you need a guide with you, in case something happens.“* [2]

*The significant others* in the study described feelings of sorrow regarding the person with dementia losing interests, losing initiative, and their increasing need for support.

*“It’s more difficult when an elderly person returns to that stage where you can’t manage, to have that level of tolerance, that it takes so much time, and you hurry up instead. As a family member it’s a really difficult procedure to accept that in some way.“* [2]

When supporting the persons with dementia, their significant others tried to be patient and compassionate, but difficulties in interpreting the persons with dementias’ wishes and the feeling of ‘nagging’ were described as burdensome. However, significant others felt empathy for the persons with dementia, and the difficulties that they were experiencing, and both significant others and persons with dementia conveyed concern for each other. Taking things ‘one day at a time’, and trying not to anticipate future sorrow was described as a way of coping. There was also a hope for a medication that could help or that deterioration would be slow.

## Category 2: using strategies to handle changes

### Building on previous habits and routines

*The persons with dementia* considered it important to maintain old habits and routines. It was still important to keep track of time, to know when something was going to happen, and to be on time for appointments. However, to continue to use the smart phone to keep track of time could be problematic, as it was often difficult to use, could be misplaced, and needed to be charged regularly; even though it was beneficial to be able to bring it outside the home.

The persons with dementia had used calendars in the past and continued to use them with varying degrees of support. Significant others often wrote upcoming events on the calendar and reminded persons with dementia to use it. The calendar was an important tool for persons with dementia to be able to participate in time management and checking for events on the calendar made it easier to keep track of what should happen when. In addition, the notes served as support for recalling events that had happened before.

*“...I appreciate it when I see that I have written it down.“* [11]

*The significant others* in the study expressed that previous lifestyle and the need to keep track of time-specific events, such as football games or news programs, affected the person with dementia’s relationship to time. Familiar habits and routines were also important. The persons with dementia were accustomed to checking the time and date on their smart phones, but it became more and more difficult. Significant others reported that the persons with dementia continued to use calendars, separate or shared, to display upcoming events and as a reminder of what had happened before, although the persons with dementia required continuous support to use them.

New strategies based on previous habits and routines were developed to deal with the person with dementia’s increasing need for support in daily time management; for example, making checklists or easy-to-understand instructions, or trying to involve the person with dementia in time management by doing it together. Starting with changes that offered positive experiences and encouraging the person with dementia to continue carrying out activities even if not correctly performed, were other examples of strategies used by significant others. However, a supporting strategy could also include providing help to prevent failure.

*“And then, of course, this disease, as soon as x gets stressed, it’s all a mess. So as a relative, as a loved one, you don’t want him to endure that, so you take the easy way out. And then most things work. At least, as we have learned to live now.“* [2]

### Better to ask than be wrong

Asking the significant other was the most common strategy described by *persons with dementia*. Questions about the time and day, whether something was going to happen, and when, were an easier alternative than checking the calendar. The persons with dementia described that they often asked questions repeatedly, either because they forgot or because they did not understand. Another reason to ask was to make sure that something was adequately perceived or performed. However, the persons with dementia also thought that repeated questions of what, when, and where could be tiresome, and described how they were trying to avoid or sift questions to relieve the strain on their significant other.

*“...and then I try to quit asking for a while and... ‘perhaps it will take care of itself’, or...but it doesn’t very often. Instead, if it’s stuck, it’s stuck. Then you finally ask. And you’ve asked three times before.“* [11]

The strategy of asking was described by *significant others* in a similar way. They were accustomed to answering frequent questions from the person with dementia, and questions about time and what would happen next were constantly recurring. However, questions about time were not necessarily perceived as burdensome:

*“...for me, it doesn’t matter, it’s more for her own sake, that she can look at the date, so she doesn’t have to ask...“* [5]

### Transferal of responsibility

*The persons with dementia* trusted that their significant other had control of everything in daily life. But while it could be a relief to let go of tasks, especially the handling of finances, it was also a process to get used to and accept their new circumstances. Moreover, there was a fear that letting go of previous activities and responsibilities would cause further deterioration. The persons with dementia also expressed concern regarding how their need for support affected their significant other, and that their spouses would get too much work, although it could be better for them to do things directly than having to correct mistakes and redo tasks.

Similarly, *significant others* described that they had gradually taken over the control of common tasks of daily life, including daily time management. Some occupations were taken over altogether, while significant others supported the persons with dementia in the performance of other tasks. The significant others provided reminders as to what should happen, when it should happen and where, and helped to initiate, sustain, adjust, and finish daily occupations in time. One of the couples described the need for support like this:

Person with dementia: *“Yes, she usually helps. Because we’re talking about me going at a certain time, for example...and I am supposed to be here and there. I don’t remember in the meantime, so I have to ask her: ‘Now what was it?’ And then she tells me. She’s cheering me on, too. Sometimes, I say, if I didn’t have her, I’d probably miss a lot of appointments.“*

Significant other: *“He wouldn’t get there at all.“*

Person with dementia: *“No, I really wouldn’t.“*

Significant other: *“You don’t get up in the morning by yourself, either. I have to wake him up, I have to plan what’s going to happen, breakfast and so on. Otherwise...“*

Person with dementia: *“Otherwise, nothing. That’s how it is, unfortunately, it’s mostly mush in my head.“* [6]

Significant others accompanied the persons with dementia to most activities outside the home. This also meant that the persons with dementia could follow what their significant others were doing. For example, when the significant other prepared for a departure, the persons with dementia noticed that it was time to start their own preparations. Since many significant others drove the persons with dementia to, for example, primary health care centres, the persons with dementia could just hang on to the significant other without having to keep track of which time they had to leave home to arrive on time for the visit. Thus, support in time management was sometimes embedded in other kinds of support.

## Category 3: time AT in daily life

### Need for professional knowledge

None of *the persons with dementia* knew about time AT before they got in contact with health care services.

*“Well, I had spoken with her, and she knew that I was... that I couldn’t keep time properly... so she said you should have one of these.“* [5]

They stressed that it was important that health care professionals took the time to understand and solve problems in daily time management. The persons with dementia that were positive regarding time AT thought that the devices should be prescribed as soon as possible.

Most *significant others* had no previous knowledge about time AT, or about the possibility of getting it prescribed. Information about suggested types of devices had been given by health care professionals during memory assessments. The significant others mentioned that there was a need for commitment on their part if the person with dementia were to receive time AT. The significant others emphasised that it was important to introduce time AT at an early stage of the dementia, because it was easier to adopt new things initially.

*“... they told us about these assistive technologies right away, but we had to call and ask for them ourselves. I think it would have been most effective if he had been given this in a box when he left. So, even though it was a tough diagnosis, he still had to consider how he will work on this.“* [2]

Information, support, and follow-ups regarding use of the time AT from healthcare professionals varied from case to case. The individual need for such interventions varied as well. Easy-to-use devices were just to start using and required no further support from healthcare services. However, more advanced time AT; ones that demanded active handling, could be difficult for significant others to at first grasp, and then introduce to the persons with dementia without support from health care professionals.

### Time AT does not replace support, but increases participation

*Persons with dementia* thought that an easy-to-use device in a specific place in the home was easy to start using, even if the significant others now and then had to remind them to use the device. This diminished the need to ask questions about time, and being able to keep track of time led to a feeling of security. However, the easy-to-use devices did not inform participants as to what was going to happen, and sometimes persons with dementia used it in combination with regular calendars.

*“Yes, but if something is particularly troublesome, I have to ask her maybe thirteen times, but then she says: “Yes, but look at the clock. What time is it and when do you have to be somewhere?“ And then I can manage to put it all together... But sometimes that doesn’t help, I have to, so to speak, someone has to show me the way.“* [6]

The persons with dementia did not think that there was a need for time AT to support time management and scheduling, as they already trusted their significant others to take care of this, but knowledge of and involvement in what was planned for the future provided a sense of security.

*Significant others* did not think that the easy-to-use electronic calendars affected their own situation to any great extent, but they appreciated that the devices supported time orientation for persons with dementia. Questions regarding time also decreased.

An advanced time AT with scheduled activities and active reminders could enhance the significant other’s feeling of security, for example that the person with dementia would remember to eat at regular times when staying home alone during the daytime. However, significant others underscored that such a device had to be introduced during an early stage in dementia, and that it may only support the person with dementia for a limited period of time. When the person with dementia used advanced time AT, the significant other first took care of the time management and administration, often by involving the person with dementia, and then provided reminders to use it. This also applied to the planning overview displayed on magnetic weekly boards.

*“And he can also call from time to time and ask ‘But when was it?’ and then you have to remind him to check. Look, it’s right there.“* [1]

Thus, advanced time AT and calendars were more of a tool for significant others to involve, communicate, and remind the person with dementia of time management by providing an overview of daily occupations for the person with dementia.

Notably, all *persons with dementia* and *significant others* would recommend their time AT to persons in similar situations.

## Discussion

This qualitative study explored the experiences of persons with dementia and significant others regarding daily time management and their perceptions on how time AT affects their everyday lives. Based on qualitative interviews with persons with dementia and significant others, the main category of “*Support by significant others is always part of daily time management”*, was found, as were the following categories: “*Facing new challenges*”, “*Using strategies to handle changes*”, and “*Time AT in daily life*”. The findings showed how important significant others are in supporting persons with dementia in daily time management. Although time AT could support orientation to time, significant others still had to place current time into context, related to the past and the future and upcoming planned events. The significant others were also increasingly responsible for the time management. But even if time AT could not completely replace support from significant others in daily time management, it could still diminish the need to ask time-related questions, facilitate participation and increase the person with dementia’s sense of independence. These are key aspects of agency, which has been defined as an individual’s capacity to initiate social action, make free choices, and influence their personal circumstances [35], which is threatened by the consequences of dementia [35, 36].

Significantly, this study found that the overall responsibility for the daily time management and planning had been transferred to significant others at an early phase of dementia. For the persons with dementia, it was experienced as both a sense of defeat in no longer being able to manage on their own, and a relief that someone you trusted took over control and made sure that things were appropriately managed. For significant others, the transferal of responsibility for the daily time management was associated with sorrow over the person with dementia’s gradual loss of independence and agency. Similar results have been reported in other research, where dementia entailed a change in occupational roles for both the persons with dementia and their significant others, leading to feelings of loss, insufficiency, and sorrow [37]. It has also been reported that persons with dementia are gradually handing over responsibility to their partner [38], and that spouses are living an intertwined occupational life, where persons with Alzheimer’s dementia become increasingly dependent on their spouse’s support for participation and occupational performance [37]. Noteworthy, a study describing and characterizing the experience and management of time and temporal problems in everyday life in dementia, found that persons with dementia living alone used a variety of strategies to manage temporal difficulties, while participants living with a spouse left the responsibility for time management to the spouse [29].

Another important finding in this study was that support in daily time management was often embedded in other kinds of support from the significant others, for example accompaniment, transport, or support in performing specific occupations. Previous research has also shown that daily adjustments and support often are masked or minimised at the onset of the dementia, to become more frequent, and later on, indispensable later on [39]. Thus, it might be difficult for persons with dementia, significant others, and health care professionals to be aware of and identify emerging time management problems, and there is a risk that time-related interventions, such as time AT for time management, will not be offered in time when the person with dementia might still be able to learn how to use the device [40]. Therefore, access to valid assessments of time-related problems is essential for health care professionals to be able to offer adequate and timely interventions, which is in accordance with previous results [4, 41].

Even when not used independently by the person with dementia, time AT and regular calendars mattered. The persons with dementia were frequently using calendars with support from their significant others; either managed by their significant others to keep the persons with dementia informed about scheduled events, or by the significant others in trying to involve the persons with dementia in time management by doing it together. In both cases, the persons with dementia needed repeated confirmation about information in the calendars. Similarly, previous research has shown that persons with dementia with memory and time management problems need continuous support from significant others in managing calendars, filling in activities and using activity reminders [27, 42]. However, support in terms of doing things together might enact agency for individuals, as agency is related to the ability to choose and act upon daily occupations and routines [43]. Thus, time AT for time management and calendars can also be a tool for enhancing agency and participation in daily life for persons with dementia; whether used independently or with support from another person. Support in orientation to time from easy-to-use time AT might also enhance the agency of the person with dementia by diminishing the need for time-related questions. Importantly, questions about time can be burdensome for significant others, but our study found that persons with dementia experienced the frequent need to ask and bother their significant others burdensome as well.

The results in this study showed that smartphones became increasingly difficult to use for persons with dementia. Although everyday technology, such as smartphones, had comparable functions as time AT devices, time AT provided an easier-to-use alternative by simplifying and clarifying needed functions. The types of time AT used in this study had gathered easily accessible information about, for example, time, date, and day/night-time on one screen in a set place, which was appreciated by participants. Research has shown that everyday technology can be difficult to use for persons with cognitive impairments [44]. One study found that development of everyday technology, such as digital strategies, services and interventions, that takes the abilities of persons with dementia into account will better contribute to an inclusive, dementia-friendly society [45]. Hence, it is still important for health care professionals to be able to offer adapted time AT for support in daily time management for persons with dementia. Furthermore, it is important that health care professionals share their knowledge on available interventions, both in terms of available time AT on prescription, and everyday technology alternatives. The participants in this study had limited knowledge on existing alternatives for supporting daily time management for persons with dementia, which is consistent with earlier findings [6]. It is also important that such information is provided at an early stage, when the person with dementia, except from better prerequisites to learn how to use a device or a new strategy, still has the need and a wish for independent daily time management. A previous study exploring the experiences of using calendar reminders from the perspective of persons with dementia and significant others found that problems and unmet needs could have been addressed by existing AT, but that unawareness of possible AT alternatives was an important barrier to AT implementation [27]. Moreover, another study found that significant others were asking for more information about technologies for persons with dementia, to be ready to act when the persons with dementia might experience a need for such support [46]. This is in line with the results in the current study, where both the persons with dementia and the significant others underscored that it was important that health care professionals understood the need for support, provided information about different solutions, and offered interventions as early as possible, with subsequent support during implementation and follow-ups based on individual needs.

### Methodological considerations

Regarding the trustworthiness of this study, some limitations must be considered. All but one interview took place during the COVID-19 pandemic, which both affected the possibility of recruiting participants and the way in which the interviews could be conducted. Interviews via telephone or video calls might be perceived as more impersonal, and it is more difficult to perceive how the other person reacts to the conversation. The interviews in this study were probably shorter and less personal than they would have been if they had been carried out face-to-face. On the other hand, it was easier to participate in a distance interview. Also, the COVID-19 restrictions, including social isolation, might have had positive effects on time availability and interest in participating in the study.

Participants in the current study described how the COVID-19 restrictions reduced the need for time management for social activities and activities outside the home, which should be noted when considering transferability. Similarly, research on adults older than 70 years of age has shown that the COVID-19 pandemic affected occupational participation and entailed changes in daily life and wellbeing [47]. Nevertheless, our study provided valuable information related to the study aim. Interviewing both persons with dementia and significant others allowed for a deeper understanding of the experience of daily time management and support related to dementia. When interviewing dyads, there might have been a risk that the persons avoided sensitive topics out of consideration for the other [48]. However, many participants openly and jointly talked about emerging problems in daily life.

To ensure trustworthiness, several methods were used. For credibility we used triangulation and peer debriefing. For transferability the population and the time context of the study were described in detail, and for confirmability the method was described in detail. Neutrality was ensured by researchers who were knowledgeable in the area, and recurrent discussions within the research group about findings and preunderstandings.

The inclusion of both persons with dementia and significant others as well as participants from both urban and rural geographical areas entailed representation of broad perspectives on daily time management in cases of dementia. In this study, more significant others (*n* = 9) than persons with dementia (*n* = 6) participated. However, including persons with dementia in research is a strength, as the perspectives of formal caregivers or significant others dominates research [25]. This study also covered the personal experiences of persons with dementia on daily time management. For transferability the reader might bear in mind that data collection was performed during the COVID-19 pandemic. In addition, it is important to consider that fourteen out of fifteen participants were spouses. Moreover, most of the significant others provided daily support, which probably affected the daily time management, including the use of time AT, for the persons with dementia.

In future research it would be of interest to include persons with dementia living alone, as well as children of persons with dementia to broaden the perspective.

## Conclusion

Significant others provide support in daily time management for persons with dementia from an early stage of the disorder. The support is often embedded in other kinds of support, which might make it more difficult to discover time management problems, while subsequent problems in time orientation appear to be more evident through frequent questions about the current time. Thus, time-related assessments and interventions compensating for time-related impairments should be offered at an early stage of dementia to increase the possibility of maintaining daily time management skills. As a time-related intervention, time AT can support orientation to time, even if significant others need to put time in context. Regarding time management, persons with dementia need increasing support from significant others. Still, whether used independently or not, time AT for time management and paper calendars might enhance the person with dementia’s agency and participation in daily occupations by facilitating involvement in time management and by providing an overview of forthcoming and past activities.

The results from this study can be used in further development of clinical methods for early identification of, and compensation for, impaired time processing ability and support in daily time management for persons with dementia. In addition, the study contributes to an increased understanding on how significant others provide support in daily time management, and the importance of providing early information about available time-related interventions. For persons with dementia without support from significant others, it is also important that society has the resources to meet the need for adequate support in daily time management.

## Data Availability

The data that support the findings of this study are available from the corresponding author upon personal request.

## Abbreviations

AT: Assistive technology
COVID-19: Coronavirus disease of 2019
MMSE: Mini-Mental State Examination
